# Growth and bioactive phytochemicals of *Panax ginseng* sprouts grown in an aeroponic system using plasma-treated water as the nitrogen source

**DOI:** 10.1038/s41598-021-82487-8

**Published:** 2021-02-03

**Authors:** Jong-Seok Song, Sunkyung Jung, Sunghoon Jee, Jung Woo Yoon, Yong Seong Byeon, Seungil Park, Seong Bong Kim

**Affiliations:** Institute of Plasma Technology, Korea Institute of Fusion Energy, Gunsan, 54004 Republic of Korea

**Keywords:** Biochemistry, Biological techniques, Physiology, Plant sciences

## Abstract

Ginseng (*Panax ginseng* Meyer) sprouts are grown to whole plants in 20 to 25 days in a soil-less cultivation system and then used as a medicinal vegetable. As a nitrogen (N) source, plasma-treated water (PTW) has been used to enhance the seed germination and seedling growth of many crops but has not been investigated for its effects on ginseng sprouts. This study established an in-situ system for N-containing water production using plasma technology and evaluated the effects of the PTW on ginseng growth and its bioactive phytochemicals compared with those of an untreated control. The PTW became weakly acidic 30 min after the air discharge at the electrodes because of the formation of nitrate (NO_3_^‒^) and nitrite (NO_2_^‒^) in the water. The NO_3_^‒^ and NO_2_^‒^ in the PTW, together with potassium ions (K^+^), enhanced the shoot biomass of the ginseng sprout by 26.5% compared to the untreated control. The ginseng sprout grown in the PTW had accumulated more free amino acids and ginsenosides in the sprout at 25 days after planting. Therefore, PTW can be used as a liquid N fertilizer for *P. ginseng* growth and phytochemical accumulation during sprouting under aeroponic conditions.

## Introduction

Ginseng (*Panax ginseng* Meyer) sprouts have been recently cultivated as a medicinal vegetable in Korea due to their relatively short period of growth in a soil-less cultivation system. Ginseng sprouts can be grown in less than 25 days after planting and still have a high content of bioactive compounds, including ginsenosides and amino acids^1–4^. Many functional and evolutionary analyses have shown that only *Panax* plants actively synthesize various ginsenosides^5–7^. Ginsenosides, showing numerous pharmacological effects in humans, including anti-inflammation^8^, highly accumulate in the ginseng shoot during the early growth stage, particularly within the first two years of planting^9,10^. In the case of other phytochemicals, non-protein amino acids are known to highly accumulate in the shoots of 1- to 3-year-old ginseng plants^1^, and they have beneficial effects against stress^11^ and immune disorders^12^.

Many efforts have been given to develop a soil-less cultivation system for ginseng plants using a nutrient solution^13,14^. In the nutrient solution, nitrogen (N) is the most important element affecting plant growth. Plants synthesize amino acids through N metabolism (NO_3_^‒^ → NO_2_^‒^ → NH_4_^+^  → Glutamine → Glutamate → Amino acids), and these amino acids are used to synthesize proteins, enzymes, and chlorophyll^15,16^. Because ginseng plants grow slowly during the early growth stage^17^, earlier application of N is essential not only for its growth but better production of bioactive phytochemicals. Previous studies reported that ginseng growth and ginsenoside synthesis were improved after N treatment through enhanced plant architecture and increased nutrient uptake in the soil^18–20^. Most commonly, synthetic N products (e.g., synthetic urea or ammonium nitrate) have been used to provide a N source for crops. The use of commercial N products requires appropriate procedures for proper storage, transportation, and disposal of the chemicals. Recent studies have reported that plasma technology is a promising tool for in-situ production of N-containing water in food and agriculture^16,21^. In an apparatus for plasma treatment, a high electric discharge produces N-containing ions in water through the dissolution of nitrogen oxides generated near the electrodes during air discharge^22–25^.

Plasma treated water (PTW) has been reported to enhance the seed germination and seedling growth of many crops but has not been investigated for its effects on ginseng sprouts in a soil-less cultivation system^26–28^. Therefore, this study was conducted to establish an in-situ system to produce N-containing water using plasma technology and to evaluate the effects of the PTW on plant growth and its bioactive phytochemicals during the early growth stage of *P. ginseng*.

## Materials and methods

### Plant materials

Two-year-old fresh *P. ginseng* was harvested from a ginseng farm located in Geumsan, Korea, in March 2020. It was washed and stored in a temperature-controlled chamber maintained at 4 °C. The *P. ginseng* rhizomes were uniformly selected based on size (9.8 ± 0.42 mm) and mass (0.9 ± 0.03 g) before the treatment.

### Plasma treated water (PTW) and aeroponic conditions

Lab-scale experiments were performed with two aeroponic systems (length 1.1 m × width 1.4 m × height 2.0 m), each equipped with a 98-L plasma treatment chamber (Supplementary Fig. S1). Deionized water inside the chambers was either untreated as the control or treated for 30 min at a distance of about 15 cm from 8 surface dielectric barrier discharge (SDBD) electrodes connected to four power supply units at the top of the lid. The same SDBD electrodes were used as in Song et al.^25^, and they generated a high electric discharge at an average power of 255 W with a driving frequency of 18 kHz and a peak-to-peak voltage of 6 kVp-p.

Plasma treated water or untreated deionized water was transferred daily into a 40-L water tank from the chamber. The plasma-treated water was adjusted to the same pH range of 6.5 ± 0.11 as the untreated deionized water with an 8.0 M potassium hydroxide (KOH) solution. Before each spraying, the *P. ginseng* rhizomes were planted in a 196-hole square plate at a distance of 5.0 × 5.0 cm from each other on the top of a 30-cm deep bath. To effectively spray the water, three of six water-distributing pipelines were placed at the bottom of the bath, and the other three were mounted at a height of 50 cm above the rhizomes. The water-distributing pipelines were equipped with spraying nozzles (Pure Water Tech., Korea) that were adjusted to deliver the water at a spray capacity of 28 mL·min^−1^ and a controlled spraying pressure of approximately 5 kgf·cm^2^. Up to 25 days after planting, the *P. ginseng* plants were repeatedly sprayed for 2 min at an interval of 58 min with the PTW or untreated deionized water in each aeroponic system maintained at 20 °C with a 24-h light. Immediately after spraying, the used water was drained from the bath.

### Ion chromatographic analysis of the PTW

Nitrogen-containing ions of the PTW or untreated deionized water were sampled immediately after air discharge for 30 min and measured using ion chromatography (Dionex ICS-2100, Thermo, USA) equipped with an IonPac AG25 column (4 × 50 mm) and an ASRS-300 (4 mm) suppressor. All the other analytical conditions were the same as those described by Song et al.^25^. Additionally, potassium ions used to adjust the pH of the PTW were measured using ion chromatography (Dionex ICS-1600, Thermo, USA) with an IonPac CG12A column (4 × 50 mm) and a CSRS-300 (4 mm) suppressor. The column temperature was 30 °C, and the suppressor was used at a current of 59 mA. Methanesulfonic acid (20 mM) as an eluent was used at a flow rate of 1.0 mL·min^−1^.

### Analysis of growth characteristics

The emergence and early growth of *P. ginseng* were evaluated up to 25 days after planting. *Panax ginseng* was regarded as emerged if its hooked stem with folded leaves was fully visible^17^. The number of emerged *P. ginseng* was counted daily for the emergence rate, and the shoot biomass was weighed for the growth rate every 5 days. Shoot emergence was expressed as a percentage of the total number of emerged shoots out of 98 *P. ginseng* rhizomes. The shoot biomass was averaged from 14 *P. ginseng* shoots harvested for each sampling date.

A logistic model was used to describe the emergence and early growth of *P. ginseng* with time after planting as follows (e.g., Shen et al.^29^; Torra et al.^30^):

$$Y= \frac{C}{1+{\left(\frac{x}{M}\right)}^{B}}$$

where $Y$ is an estimate of the cumulative shoot emergence (%) or shoot biomass (g·plant^−1^) at days ($x$) after planting; $C$ is the maximum shoot emergence or shoot biomass at which the lag time is infinite; $B$ is the rate of increase of the shoot emergence or shoot biomass, and $M$ is the time lag to reach 50% of the maximum cumulative shoot emergence or shoot biomass. The goodness of fit of the model to the data was assessed with the adjusted R^2^.

### Analysis of amino acids

*Panax ginseng* plants were harvested and divided into shoots and roots at 25 days after planting. Each part was pooled and lyophilized at a temperature below − 70 °C and then hydrolyzed with 6.0 M hydrochloric acid (HCl) at 110 °C for 22 h. The hydrolysate was dried at 50 °C, dissolved in 0.02 M HCl, and filtered through a 0.45-μm syringe filter (Whatman) before analysis. The amino acid analysis was performed using an amino acid analyzer (Hitachi L-8900, Hitachi High-Technologies Co., Japan). The column used was a Hitachi HPLC packed column with ion exchange resin (No. 2622PF, 4.6 × 60 mm). A series of Kanto L-8900 buffer solutions (PF-1, 2, 3, 4 and RG, Kanto Chemical Co., Japan) were used as the mobile phase. A calibration curve method was used to quantify the 38 free amino acids in the sample. A standard amino acid mixture (Type B & Type AN-2, Wako Pure Chemical Industries, Japan) included the following: 8 essential amino acids that were threonine, methionine, lysine, histidine, valine, isoleucine, leucine, and phenylalanine, and 30 non-essential amino acids that were phosphoserine, phosphoethanolamine, urea, aspartic acid, alanine, 2-aminoethanol, ornithine, arginine, hydroxyproline, proline, taurine, serine, glutamic acid, α-aminoadipic acid, sarcosine, glycine, citrulline, α-aminobutyric acid, cysteine, cystathionine, tyrosine, β-alanine, β-aminoisobutyric acid, γ-aminobutyric acid, ammonia, hydroxylysine, 1-methylhistidine, 3-methylhistidine, anserine, and carnosine. All the calibration curves showed good linearity.

### Analysis of ginsenosides

The shoots and roots of the *P. ginseng* were separately harvested every 5 days. Each part was pooled and lyophilized at a temperature below − 70 °C and then ground to a powder. For each sample, a 0.5 g aliquot was added to 25 mL of 80% aqueous methanol and extracted using an ultrasonicator for 2 h at 40‒50 °C. The extract was centrifuged at 13,000 rpm for 10 min, and the supernatant was filtered through a 0.45-μm syringe filter (Whatman) before analysis. Ginsenoside analysis was performed by HPLC (Agilent Technologies 1260, USA) equipped with a diode array detector. The column used was a reversed-phase column (Zorbax Eclipse Plus C18, 4.6 × 150 mm, 3.5 µm), and the column temperature was 30 °C. The mobile phase was a gradient of deionized water (A) and acetonitrile (B): 20% B (0‒11 min), 20‒30% B (11‒22 min), 30‒50% B (22‒50 min), 50% B (50‒70 min), 50‒70% B (70‒80 min), and 20% B (80‒90 min). The flow rate of the mobile phase was 1.0 mL·min^−1^, and the absorbance of the ginsenosides was measured at 203 nm. A calibration curve method was used to quantify the 18 ginsenosides in the sample. The ginsenoside standards (Ambo Institute, Korea) were prepared in methanol. They included 7 protopanaxadiol (PPD)-type ginsenosides (Rb1, Rb2, Rb3, Rc, Rd, Rg3, and Rh2), 5 protopanaxatriol (PPT)-type ginsenosides (Re, Rf, Rg1, Rg2, and Rh1), and 6 other ginsenosides (Rg5, Rg6, Rh4, Rk1, Rk3, and F4). All the calibration curves showed good linearity.

### Statistical analysis

All the data were initially subjected to analysis of variance, and a mean comparison was made by Tukey’s HSD (honestly significant difference) test at *P* = 0.05. Data from each sampling date were analyzed with the treatment as a fixed factor and the replicate as a random factor. All the statistical analyses were done with Origin Pro 8.0 (Origin Lab Co., Northampton, MA, USA).

## Results and discussion

### Formation of nitrogen-containing ions in the PTW

The chemical properties of the water were evaluated immediately after air discharge for 30 min. The water became weakly acidic during the plasma treatment; therefore, it was adjusted to a pH of 6.6 ± 0.34 for the *P. ginseng* growth using a KOH solution. The acidity of the PTW was accompanied by the formation of nitrate (NO_3_^‒^) and nitrite (NO_2_^‒^) in the water. The concentration of the NO_3_^‒^ and NO_2_^‒^ in the PTW was 5.2 and 0.1 mg·L^−1^, respectively, while the K^+^ concentration was 5.0 mg·L^−1^ (*P* < 0.05) (Table 1). As previously reported, NO_3_^‒^ and NO_2_^‒^ can be formed in PTW through the dissolution of nitrogen oxides (NO, NO_2_, and N_2_O_3_) formed near the electrodes when a high electric discharge is applied to air^22–25^. The dissolution of the nitrogen oxides in the water contributes to the low pH of the PTW, which can be explained by the reaction between NO_2_ and H_2_O (2NO_2_ + H_2_O → NO_2_^‒^ + NO_3_^‒^ + 2H^+^)^16^. Our results thus indicate that plasma treatment can produce N-containing ions in water. The N-containing ions in the PTW can be used to supply a N source to *P. ginseng* plants. In particular, the NO_3_^‒^ concentration of 5 mg·L^−1^ might be sufficient for *P. ginseng* growth during sprouting. The KNO_3_ at similar concentration has been previously used as a macro nutrient for *P. ginseng* plants under hydroponic conditions^13,14^.

**Table 1:** Ion concentration of the plasma-treated water (PTW) containing potassium ions (K^+^) compared with the untreated deionized water (DW).

Sample	Nitrite (mg·L^−1^)	Nitrate (mg·L^−1^)	Potassium (mg·L^−1^)
DW	0	0	0
PTW + K^+^	0.1 ± 0.01*	5.2 ± 0.27*	5.0 ± 1.37*

*An asterisk indicates a significant difference between the untreated deionized water and plasma-treated water (*P* < 0.05).

### Ginseng growth after spraying K^+^-containing PTW

**Figure 1:**
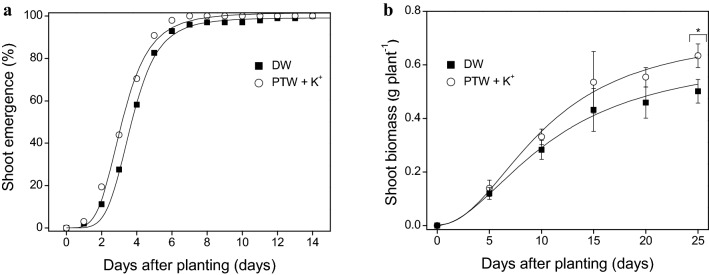
Shoot emergence (**a**) and shoot biomass (**b**) of *Panax ginseng*, each sprayed with deionized water (DW) and plasma-treated water (PTW) containing potassium ions (K^+^) up to 25 days after planting. The shoot emergence was expressed as a percentage of the total number of emerged shoots out of 98 *P. ginseng* rhizomes. The shoot biomass was averaged from 14 *P. ginseng* shoots harvested for each sampling date. An asterisk indicates a significant difference between the DW and PTW + K^+^ at each day (*P* < 0.05).

The emergence and early growth of *P. ginseng* were accurately described by the logistic model (Fig. 1). The shoot of *P. ginseng* sigmoidally emerged and grew vigorously during sprouting; conversely, the root biomass decreased (Fig. 1; Supplementary Fig. S2). For the shoot emergence*,* the maximum value and time to reach 50% of the maximum value were estimated to be 99.5% and 3.6 days for the untreated control and 101.8% and 3.1 days for the *P. ginseng* sprayed with the K^+^-containing PTW for 14 days (Table 2). The maximum shoot emergence showed little or no difference between the untreated control and the *P. ginseng* sprayed with the K^+^-containing PTW (Fig. 1a). The time required for 50% of the maximum shoot emergence was approximately 0.5 days earlier when sprayed with the K^+^-containing PTW compared to the untreated control. For the shoot biomass, the maximum value and time to reach 50% of the maximum value were estimated to be 0.64 g·plant^−1^ and 11.0 days for the untreated control and 0.72 g·plant^−1^ and 10.3 days for the *P. ginseng* sprayed with the K^+^-containing PTW for 25 days (Table 2). At 25 days after planting, the actual shoot biomass showed a significant difference between the *P. ginseng* sprayed with the K^+^-containing PTW and the untreated control (*P* < 0.05) (Fig. 1b). The shoot biomass of the *P. ginseng* sprayed with the K^+^-containing PTW was 26.5% higher than that of the untreated control. The time required for 50% of the maximum shoot biomass was approximately 0.7 days earlier for the *P. ginseng* sprayed with the K^+^-containing PTW compared to the untreated control (Table 2). These results indicate that *P. ginseng* can emerge faster and grow rapidly with the K^+^-containing PTW for 25 days. The N and K in the PTW are major macro elements affecting plant growth; for example, NO_3_^‒^ is consequently reduced to amino acids which can be used to synthesize proteins, enzymes, and chlorophyll^16^, and K maintains cation–anion balance within the plant^31^. Previous studies reported that K^+^-containing PTW enhanced the seed germination and seedling growth of crops due to the N and K supply to the crops. The seeds of sweet basil and spinach grew more rapidly into seedlings after being sprayed with K^+^-containing PTW^31,32^. Thus, N in the PTW, together with K, could be used as main constituents for plant growth.

**Table 2:** Parameter estimates for the logistic model for the regression of the *Panax ginseng* shoots, each sprayed with deionized water (DW) and plasma-treated water (PTW) containing potassium ions (K^+^) up to 25 days after planting.

	Shoot emergence (%)				Shoot biomass (g·plant^−1^)			
	$C$^1^	$B$^2^	$M$^3^	R^2^	$C$	$B$	$M$	R^2^
DW	99.5 (0.89)	− 4.6 (0.27)	3.6 (0.05)	0.99	0.64 (0.081)	− 1.9 (0.37)	11.0 (1.87)	0.99
PTW + K^+^	101.8 (1.07)	− 3.8 (0.26)	3.1 (0.06)	0.99	0.72 (0.089)	− 2.1 (0.47)	10.3 (1.61)	0.99

^1^$C$ represents the maximum shoot emergence or shoot biomass.

^2^$B$ is the rate of increase of the shoot emergence or shoot biomass.

^3^$M$ is the time lag to reach 50% of the maximum cumulative shoot emergence or shoot biomass.

### Amino acid contents after spraying *P. ginseng* with the K^+^-containing PTW

**Figure 2:**
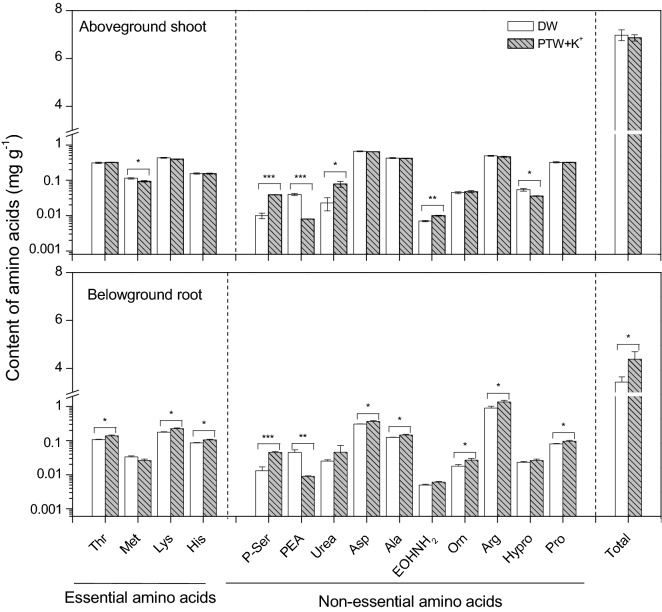
Total contents of the 14 free amino acids of *Panax ginseng* at 25 days after planting, each sprayed with deionized water (DW) and plasma-treated water (PTW) containing potassium ions (K^+^) up to 25 days. The fourteen free amino acids include 4 essential amino acids (threonine (Thr), methionine (Met), lysine (Lys), and histidine (His)) and 10 non-essential amino acids (phosphoserine (*P*-Ser), phosphoethanolamine (PEA), urea, aspartic acid (Asp), alanine (Ala), 2-aminoethanol (EOHNH_2_), ornithine (Orn), arginine (Arg), hydroxyproline (Hypro), and proline (Pro)). Each bar represents the mean of four replicates with each replicate containing three shoots (or roots). The error bars represent the standard error of that mean. An asterisk indicates a significant difference between the DW and PTW + K^+^ at 25 days (*P* < 0.05).

Nitrogen-containing ions produced in the PTW were involved in the enhanced free amino acid contents in the *P. ginseng* sprouts. Spraying with the K^+^-containing PTW increased the total free amino acid contents by 27.7% in the *P. ginseng* root compared to the untreated control (*P* < 0.05) (Fig. 2; Supplementary Table S1). In particular, 3 essential amino acids (threonine, lysine, and histidine) and 6 non-essential amino acids (phosphoserine, aspartic acid, alanine, ornithine, arginine, and proline) were increased significantly (*P* < 0.05). Arginine and asparagine (derivatives of aspartic acid) have been reported to be used as N storage and transport compounds in ginseng roots^1,33,34^. Threonine, lysine, and alanine are derived from aspartic acid in amino acid catabolic pathways in plants^34,35^. Phosphoserine is an intermediate in the production of threonine^35^. Arginine can be converted to proline via ornithine^36^. Thus, *P. ginseng* sprouts might accumulate excess N as arginine and aspartic acid in roots and consecutively, convert them into their related amino acids. Additionally, the *P. ginseng* plant might be exposed to some stress due to the K^+^-containing PTW sprayed for 25 days. Of the related amino acids, proline acts as an osmolyte and a chemical chaperone and accumulates in plants under various stress conditions^34^. Moreover, histidine has an important role as an antioxidant in free radical scavenging under stress conditions^37–39^.

After spraying *P. ginseng* with the K^+^-containing PTW for 25 days, the non-essential amino acid phosphoethanolamine was significantly decreased in the root (*P* < 0.05) (Fig. 2). In our study, the *P. ginseng* sprout grew in the absence of added phosphorus (P), although N and K were readily available for rapid growth. For *P. ginseng* growth, the P-starved root might require some metabolism of P-containing proteins to cope with the P starvation. Under a P-starved condition, a slight but significant reduction of phosphoethanolamine was detected in Arabidopsis roots; this result indicates that phosphoethanolamine can be dephosphorylated in the phospholipids under P starvation^40,41^.

Spraying the *P. ginseng* with the K^+^-containing PTW had little effect on the total free amino acid contents of the shoot but significantly affected the composition of the free amino acids compared with the untreated control (Fig. 2). After spraying the *P. ginseng* with the K^+^-containing PTW for 25 days, 3 non-essential amino acids (phosphoserine, urea, and 2-aminoethanol) were significantly increased in the shoot (*P* < 0.05). In contrast, another 2 non-essential amino acids (phosphoethanolamine and hydroxyproline) and the essential amino acid methionine were decreased significantly (*P* < 0.05). Urea acts as N storage and a long-distance transport compound in many plants^34^. 2-aminoethanol functions as a signal for initiating stress tolerance and serves as a membrane stabilizer^42^. Hydroxylation of proline to hydroxyproline normally occurs during its deposition in the cell walls, indicating that a deficiency of hydroxyproline in cell wall proteins can be related to some stress conditions^43–45^. The *P. ginseng* sprouts might respond to the P deficiency in our study even though N and K were readily available for rapid growth. Under the P-starved condition, metabolic conversion of phosphoethanolamine to 2-aminoethanol occurs in the phospholipids to cope with the P starvation^40,41^. Following the decrease of phosphoethanolamine, methionine might be required less in *P. ginseng* sprouts as a methyl group donor for phosphoethanolamine. The methyl groups originating from methionine are utilized to methylate phosphoethanolamine to various phosphoethanolamine derivatives involved in the synthesis of phospholipids^46^.

### Ginsenoside contents after spraying* P. ginseng* with the K^+^-containing PTW

**Figure 3:**
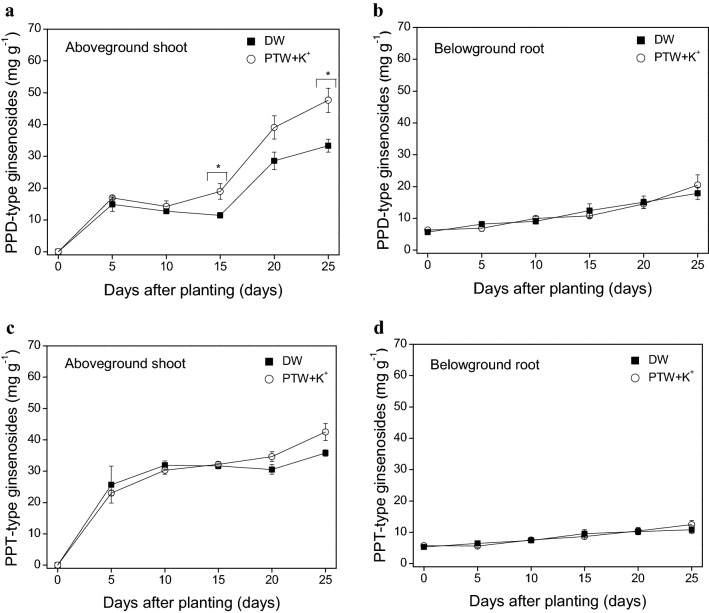
Protopanaxadiol (PPD)-type (**a**,**b**) and protopanaxatriol (PPT)-type (**c**,**d**) ginsenosides in the aboveground shoot (**a**,**c**) and belowground root (**b**,**d**) of *Panax ginseng*, each sprayed with deionized water (DW) and plasma-treated water (PTW) containing potassium ions (K^+^) for 25 days. Each symbol represents the mean of four replicates with each replicate containing three shoots (or roots). The error bars represent the standard error of that mean. An asterisk indicates a significant difference between DW and PTW + K^+^ at each day (*P* < 0.05).

Regardless of the PTW treatment, the total content of the 18 ginsenosides increased more rapidly in the shoot than in the root up to 25 days after planting the *P. ginseng* in the aeroponic system (Supplementary Fig. S3). Spraying with the K^+^-containing PTW increased the total ginsenoside contents by 30.0% in the *P. ginseng* shoot compared to the untreated control at 25 days (*P* < 0.05). Two types of ginsenosides accounted for more than 90% of the total ginsenosides: 7 PPD-type ginsenosides (Rb1, Rb2, Rb3, Rc, Rd, Rg3, and Rh2) and 5 PPT-type ginsenosides (Re, Rf, Rg1, Rg2, and Rh1) (Fig. 3; Supplementary Fig. S3). The ratio of PPD-type to PPT-type ginsenosides increased from 0.6 to 1.0 and from 1.0 to 1.6 in the *P. ginseng* shoot and root up to 25 days after planting, respectively (Fig. 3). These results correspond to the previous findings of Kim et al.^47^. The PPD-type and PPT-type ginsenosides are highly produced in the shoot during the early growth stage of 3-year-old *P. ginseng*. The ratio of PPD-type (Rb1, Rb2, Rc, and Rd) to PPT-type (Re, Rg1, and Rh1) ginsenosides is always lower in the shoot than in the root^47^. The PPD-type/PPT-type ratio is an important factor for the pharmacological efficacy of *P. ginseng*^48^. The low PPD-type/PPT-type ratio of *P. ginseng* has been reported to enhance neurocognitive function^48^. Thus, *P. ginseng* shoots can be pharmacologically beneficial for human health.

**Figure 4:**
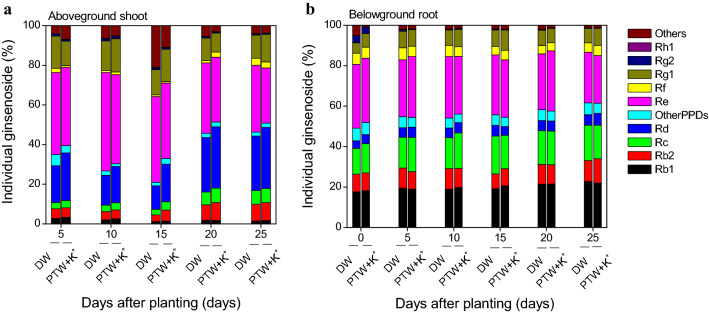
The percentages of individual ginsenosides in the aboveground shoot (**a**) and belowground root (**b**) of *Panax ginseng*, each sprayed with deionized water (DW) and plasma-treated water (PTW) containing potassium ions (K^+^) for 25 days.

The composition of individual ginsenosides differed in the different parts of *P. ginseng* (Fig. 4). Three PPD-type ginsenosides (Rb1, Rb2, and Rc) accounted for approximately 7.3‒18.1% of the total ginsenosides in the shoot, while Rd accounted for approximately 11.9‒30.9%; in the root, Rb1, Rb2, and Rc accounted for approximately 39.1‒50.6% and Rd for approximately 3.8‒5.8%. Two PPT-type ginsenosides Re and Rg1 accounted for approximately 41.8‒64.5% and 32.0‒38.5% of the total ginsenosides in the shoot and the root, respectively. The difference in the composition of each part of *P. ginseng* might be attributed to the movement of individual ginsenosides from the root to the shoot, or vice versa during the early growth stage. Two PPT-type ginsenosides Re and Rg1 seem to be preferentially synthesized and stored in the shoot, while three PPD-type ginsenosides Rb1, Rb2, and Rc seem to be preferentially stored in the root^47,49^. The PPT-type ginsenosides Re and Rg1 exert anti-inflammatory effects^8^, and the PPD-type ginsenosides Rb1, Rb2, and Rc stimulate immune responses^50^. Thus, the whole plant of *P. ginseng* can be used as a medicinal vegetable.

When *P. ginseng* was sprayed with the K^+^-containing PTW, the total content of PPD-type ginsenosides were increased more in the shoot compared to the untreated control (Fig. 3a). Spraying the *P. ginseng* with the K^+^-containing PTW increased the content of the PPD-type ginsenosides by about 1.7-fold compared to the untreated control at 15 days (*P* < 0.05). The higher content of PPD-type ginsenosides was extended for 10 days (*P* < 0.05). In particular, ginsenoside Rd was the most abundant PPD-type ginsenoside in the shoot (Fig. 4a). The ginsenoside Rd accounted for approximately 24.6% and 20.1% of the total ginsenosides in the treated and untreated shoot, respectively; 3 ginsenosides Rb1, Rb2, and Rc accounted for approximately 13.9% and 12.1%. Conversely, 3 ginsenosides Rb1, Rb2, and Rc were the most abundant PPD-type ginsenosides in the root (Fig. 4b). Three ginsenosides Rb1, Rb2, and Rc accounted for approximately 46.1% and 45.3% of the total ginsenosides in the treated and untreated root, respectively; ginsenoside Rd accounted for approximately 4.9% and 4.8%. In the case of the PPT-type ginsenosides, there was little or no significant difference between the *P. ginseng* sprayed with the K^+^-containing PTW and untreated control in the shoot and root (*P* > 0.05) (Fig. 3c,d). Among the 5 PPT-type ginsenosides, ginsenoside Re was the most abundant PPT-type ginsenoside regardless of the different parts of *P. ginseng* (Fig. 4). The ginsenoside Re accounted for approximately 36.6% and 40.7% of the total ginsenosides in the treated and untreated shoot, respectively, and approximately 28.7% and 28.8% in the treated and untreated root. Thus, our results indicate that spraying with K^+^-containing PTW can affect the production of PPD-type ginsenosides in the shoot, especially ginsenoside Rd.

To our knowledge, information about the change in individual ginsenosides versus KNO_3_ during the whole-plant growing period has not been reported much. In our study, the enhanced content of the PPD-type ginsenosides by KNO_3_ could be related to the increased transcription of ginsenoside biosynthesis-related genes. A similar study with KNO_3_ reported an enhanced saponin content in suspension cultures of *P. ginseng*^51^. Later, transcriptomic analysis revealed that a similar concentration (about 5 mg·L^−1^) of KNO_3_ could enhance the transcript levels of genes associated with ginsenoside biosynthesis under cold stress^52,53^. In addition, the concentration of KNO_3_ can enhance the expression of genes encoding antioxidant enzymes and the activities of corresponding enzymes^53^. Potassium seems to be a major contributor to oxidative stress tolerance by activating antioxidant enzymes^53^ and increasing ginsenoside production^51^. Therefore, the enhanced content of ginsenoside Rd could be reasonably expected under stress conditions (e.g., P deficiency) because it exerts antioxidant activities^54^.

### The use of PTW for growth and bioactive phytochemicals

Plasma treated water can supply a N source to *P. ginseng* plants during the early growth stage. The PTW, which can have a broad concentration of N-containing ions, is effective as a liquid N fertilizer. A low concentration (about 5 mg·L^−1^) of NO_3_^‒^ can be used for *P. ginseng* growth during sprouting under aeroponic conditions. In the case of bioactive phytochemicals, the NO_3_^‒^ in the PTW, together with K^+^, can significantly help ginseng plants accumulate free amino acids and ginsenosides, although the detailed mechanisms have not been investigated yet.

The changes in bioactive phytochemicals depend on biological (e.g., organ, growth and development stages, and age)^2,9,47,55,56^ and environmental factors (e.g., stress, light quality, and cultural practices)^4,14,19,49,57,58^. In our study, NO_3_^‒^ in the PTW, together with K^+^, was involved in the enhanced free amino acid and ginsenoside contents in the *P. ginseng* sprout. Nitrogen significantly affects ginsenoside biosynthesis, although ginsenosides are non-N-containing metabolites^20,59,60^. Later, the variations in different organs have been mainly attributed to the movement of individual phytochemicals from the root to the shoot or vice versa throughout the growing season^1,47^. Further studies are needed to understand better the biosynthesis and accumulation of individual phytochemicals in *P. ginseng* sprouts treated with K^+^-containing PTW.

## Supplementary Information

Supplementary Information
