# Supplementary material for: Growth and bioactive phytochemicals of Panax ginseng sprouts grown in an aeroponic system using plasma-treated water as the nitrogen source
Source: Sci Rep. 2021 Feb 3;11:2924. doi: 10.1038/s41598-021-82487-8 (PMC7859182; doi:10.1038/s41598-021-82487-8)
Supplement: Supplementary file 1 — Supplementary Information [file 41598_2021_82487_MOESM1_ESM.docx]

Growth and bioactive phytochemicals of *Panax ginseng* sprouts grown in an aeroponic system using plasma-treated water as the nitrogen source

Jong-Seok Song^*^, Sunkyung Jung, Sunghoon Jee, Jung Woo Yoon, Yong Seong Byeon, Seungil Park, Seong Bong Kim

Institute of Plasma Technology, Korea Institute of Fusion Energy, Gunsan 54004, Republic of Korea

*** Corresponding author:**Jong-Seok Song
[jongseoksong@kfe.re.kr](mailto:jongseoksong@kfe.re.kr)

**
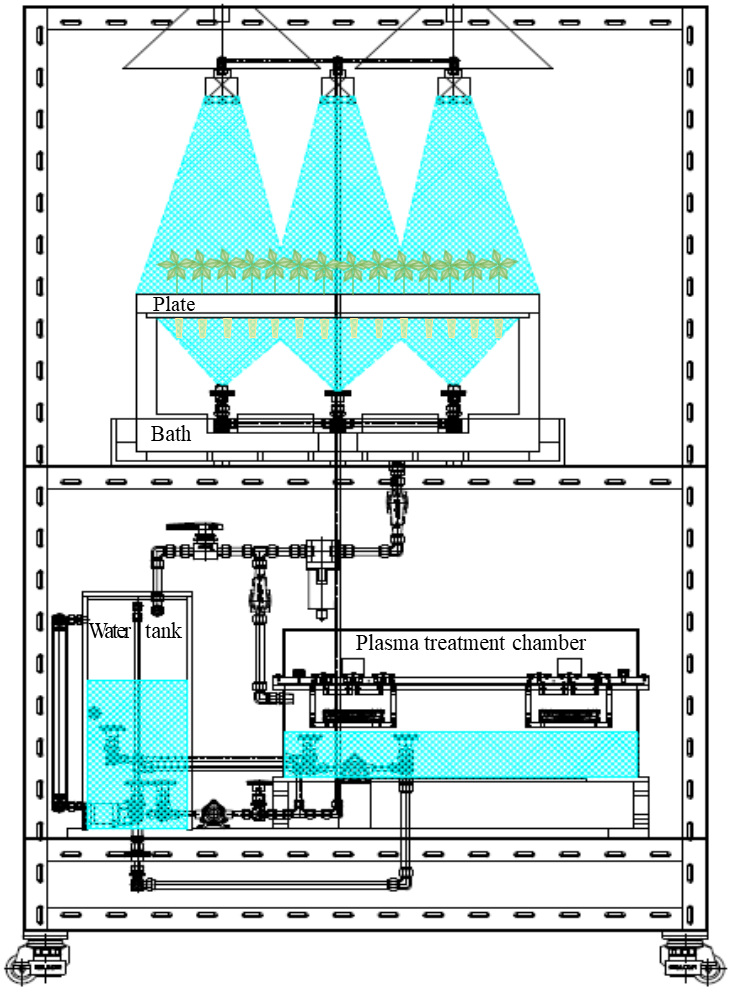
**

**Supplementary Figure S1.** Schematic diagram of the aeroponic system equipped with a 98-L plasma treatment chamber (Institute of Plasma Technology, Korea Institute of Fusion Energy, Republic of Korea).

**Supplementary Figure S2.** Root biomass of *Panax ginseng*, each sprayed with deionized water (DW) and plasma-treated water (PTW) containing potassium ions (K^+^) up to 25 d after planting. The root biomass was averaged from 14 roots of *P. ginseng* harvested for each sampling date.

**a b**

**Supplementary Figure S3.** Total contents of the 18 ginsenosides in the aboveground shoot (**a**) and belowground root (**b**) of *Panax ginseng*, each sprayed with deionized water (DW) and plasma-treated water (PTW) containing potassium ions (K^+^) for 25 d. Each symbol represents the mean of four replicates with each replicate containing three shoots (or roots). The error bars represent the standard error of that mean. An asterisk indicates a significant difference between the DW and PTW+K^+^ at each day (*P* < 0.05).

**Supplementary Table S1.** Contents of the 24 amino acids of *Panax ginseng* at 25 days after planting, each sprayed with deionized water (DW) and plasma-treated water (PTW) containing potassium ions (K^+^) up to 25 d.

|  | Aboveground shoot | |  | Belowground root | |
| --- | --- | --- | --- | --- | --- |
| Amino acid  (mg g^-1^) | DW | PTW+K^+^ |  | DW | PTW+K^+^ |
| Essential  amino acids |  |  |  |  |  |
| Valine | 0.523 (0.0213) | 0.527 (0.0268) |  | 0.157 (0.0046) | 0.185 (0.0126) |
| Isoleucine | 0.284 (0.0137) | 0.282 (0.0175) |  | 0.095 (0.0036) | 0.111 (0.0084) |
| Leucine | 0.645 (0.0242) | 0.631 (0.0116) |  | 0.191 (0.0081) | 0.224 (0.0145) |
| Phenylalanine | 0.403 (0.0141) | 0.398 (0.0110) |  | 0.125 (0.0075) | 0.151 (0.0098) |
| Non-essential  amino acids |  |  |  |  |  |
| Taurine | 0.003 (0.0006) | N.D. |  | 0.004 (0.0035) | N.D. |
| Serine | 0.319 (0.0126) | 0.315 (0.0092) |  | 0.100 (0.0042) | 0.112 (0.0032) |
| Glutamic acid | 0.879 (0.0306) | 0.821 (0.0169) |  | 0.530 (0.0382) | 0.636 (0.0470) |
| α-Aminoadipic acid | N.D. | N.D. |  | 0.003 (0.0004) | 0.001 (0.0004) |
| Sarcosine | N.D. | N.D. |  | N.D. | N.D. |
| Glycine | 0.375 (0.0106) | 0.373 (0.0083) |  | 0.096 (0.0018) | 0.111 (0.0057) |
| Citrulline | N.D. | N.D. |  | 0.001 (0.0002) | 0.002 (0.0006) |
| α-Aminobutyric acid | 0.001 (0.0004) | 0.001 (0.0006) |  | 0.002 (0.0004) | 0.002 (0.0007) |
| Cysteine | 0.047 (0.0011) | 0.044 (0.0027) |  | 0.014 (0.0020) | 0.020 (0.0015) |
| Cystathionine | 0.003 (0.0007) | 0.003 (0.0011) |  | 0.002 (0.0002) | 0.003 (0.0006) |
| Tyrosine | 0.240 (0.0088) | 0.237 (0.0012) |  | 0.044 (0.0058) | 0.051 (0.0033) |
| β-Alanine | 0.004 (0.0007) | 0.007 (0.0013) |  | N.D. | N.D. |
| β-Aminoisobutyric acid | 0.004 (0.0008) | 0.001 (0.0007) |  | 0.002 (0.0010) | 0 |
| γ-Aminobutyric acid | 0.054 (0.0057) | 0.046 (0.0022) |  | 0.054 (0.0059) | 0.058 (0.0052) |
| Ammonia | 0.086 (0.0029) | 0.080 (0.0054) |  | 0.069 (0.0027) | 0.074 (0.0060) |
| Hydroxylysine | N.D. | N.D. |  | N.D. | N.D. |
| 1-Methylhistidine | N.D. | N.D. |  | N.D. | N.D. |
| 3-Methylhistidine | N.D. | N.D. |  | N.D. | N.D. |
| Anserine | N.D. | N.D. |  | N.D. | N.D. |
| Carnosine | N.D. | N.D. |  | N.D. | N.D. |

All values are expressed as the mean ± standard error (n=4).

N.D. represents not detected.
